# Physiological differences in cardiopulmonary exercise testing between children and adults

**DOI:** 10.1038/s41390-025-04212-9

**Published:** 2025-06-19

**Authors:** Valentina Papic, Romina Ledergerber, Ralf Roth, Raphael Knaier

**Affiliations:** Department of Sport, Exercise and Health, University of Basel, Basel, Switzerland

## Abstract

**Background:**

Physiological responses to exercise differ between children and adults, but achieving maximal exertion in children complicates the interpretation of VO_2__max_. This study, therefore, examines age- and sex-related physiological differences in submaximal parameters during incremental exercise.

**Methods:**

In this cross-sectional study, 24 children (7–11 years), 20 moderately trained adults (MTA), and 20 well-trained adults (WTA; 20–30 years) completed a maximal incremental exercise test on a cycle ergometer with continuous respiratory measurement. Linear regression models analysed age and sex differences in ventilatory thresholds (VT1, VT2) and oxygen uptake efficiency slope and plateau (OUES), with Cohen’s *d* effect sizes reported.

**Results:**

Children showed higher body mass-adjusted VO_2_ at VT1 and VT2 (*d* = 0.58–0.66) compared to MTA, and slightly lower VT2 values than WTA (*d* = 0.35). Adults had higher absolute OUES (*d* = 0.37–1.45) and OUEP (*d* = 0.60–0.81), while children exhibited higher body mass-adjusted OUES (*d* = 0.87 – 1.80). Males had higher VO_2_ at VT2, OUES, and OUEP (*d* = 0.41–0.81), while females showed higher relative VO_2_ at VT1 and VT2 (*d* = 0.44–0.59) compared to males.

**Conclusions:**

Children rely more on oxidative metabolism than adults. Maturation influences exercise efficiency more than body mass, underscoring physiological differences. These age- and sex-specific patterns call for longitudinal studies to further explore the roles of growth and training.

**Impact:**

- This study identifies clear physiological differences in submaximal CPET parameters between children and adults.
- It adds novel insight by including both ventilatory thresholds and oxygen uptake efficiency, adjusted for body mass and training status.
- The findings suggest children rely more on oxidative metabolism, emphasizing the importance of maturation on exercise efficiency and informing age- and sex-specific assessment protocols in pediatric exercise physiology.

## Introduction

Cardiopulmonary exercise testing (CPET) serves as an important method to examine the physiological reactions of the pulmonary, cardiovascular, and metabolic systems to exercise.^1^ The most frequently used outcome in CPET is the maximum oxygen uptake (VO_2max_) as it represents aerobic capacity. However, a significant drawback of this parameter is that it requires maximum exercise effort for accurate interpretation, for which high motivation is a prerequisite.^2^ Furthermore, even if an individual is highly motivated, identifying true VO_2max_ is especially challenging in children due to missing valid criteria for maximal exhaustion.^3^ The lack of valid criteria might be due to physiological differences in response to exercise between children and adults. These differences include a greater oxidative yet diminished anaerobic energetic response,^4^ an increased dependence on fat as primary fuel source^5^ and potentially a greater proportion of type I muscle fibers in children.^6,7^ In adults, robust secondary criteria derived from VO_2_ plateaus^8–10^ have been established for the determination of true VO_2max_.^11,12^ In contrast, secondary exhaustion criteria in children are based on somewhat arbitrary values and are not data-driven.^13^ This might be due to the lower incidence of VO_2_ plateau in children compared to adults, possibly reflecting the lower anaerobic capacity, making it difficult to establish robust criteria.

Thus, to investigate physiological differences in the response to exercise between adults and children, it seems more promising to explore submaximal exercise parameters,^1^ namely ventilatory thresholds (VTs) and the oxygen uptake efficiency (OUE). These parameters offer important information about the transition from aerobic to anaerobic metabolism, as well as the efficiency of the system, respectively. Although some differences between children and adults have been identified in these parameters, they have generally received limited attention in the past.

### Ventilatory thresholds

The first VT (VT1) is a key indicator of aerobic exercise capacity^14^ and marks the transition from light to moderate intensity exercise.^15^ At this point, pulmonary ventilation (volume expiration [VE]) begins to rise proportionally compared to oxygen consumption (VO_2_), without a corresponding increase in VE relative to carbon dioxide production (VCO_2_).^16^ Some cross-sectional studies have compared VO_2_ relative to body mass at VT1 (VO_2_ at VT1) and VO_2_ at VT1 relative to VO_2max_ (VT1/VO_2max_) across different age groups, demonstrating a tendency for both to decline with age.^17,18^ This decline indicates that with increasing age, children tend to reach their VT1 at a lower percentage of their VO_2max_. This reduction could be related to the change from a strong oxidative metabolism to an increased dependence on glycolytic metabolic pathways during maturation,^6^ or methodologically to the possibly increasing ability of the subjects to reach maximum exhaustion with maturation. Additionally, Reybrouck et al.^18^ found that girls reached VT1 at a significantly lower VO_2_ than boys of the same age. As VO_2max_ could not be validly determined due to the lack of exhaustion criteria in these studies,^17,18^ the results of the relative differences in VT1/VO_2max_ should be interpreted with caution and require further investigation.

The second VT (VT2) is identified as the point between moderate and high intensity exercise where there is a notable exponential rise in VE in relation to VO_2_ and VE in relation to VCO_2_. This point marks the transition to a substantial increase in anaerobic metabolism.^19^ It is a strong indicator of a person’s capability to perform vigorous physical activity for longer durations.^20^ This parameter would be highly interesting considering the role of anaerobic metabolism in explaining the physiological differences between children and adults, but unfortunately, VT2 has hardly been studied in children.^14^

### Oxygen uptake efficiency

The Oxygen Uptake Efficiency Slope (OUES)^21^ and the Oxygen Uptake Efficiency Plateau (OUEP) offer a submaximal assessment of the cardiorespiratory system’s function throughout incremental exercise.^22^ The measurement of OUE, defined as the inverse of the ventilatory equivalent for oxygen (VE/VO_2_), estimates how effectively ventilation (VE) supports oxygen uptake (VO_2_). The concept of the OUE slope (OUES) arises from the nonlinear relationship between VE and VO_2_ during an incremental CPET,^21^ while OUEP represents the point of maximum efficiency during the test. Higher OUES values indicate greater efficiency in oxygen consumption, while elevated OUEP values reflect improved oxygen utilization. Conversely, lower values signify less oxygen consumption for a given level of ventilation efficiency.^22^

As maturation brings changes in height, body mass, and body composition, resulting in alterations in respiratory function, it is expected that absolute peak values of both VO_2_ and VE will also change, likely influencing OUES.^2^ Indeed, Bongers et al.^22^ reported that both the OUEP and the OUES increase with age in both boys and girls, with boys achieving higher OUES values than girls. Rogowski et al.^23^ found that absolute OUES values were higher in more mature ones compared to less mature groups, implying that with maturation, the human body develops greater efficiency in oxygen consumption.

As there are very few studies using different methods to investigate OUES and OUEP in children, the differences in efficacy are still unclear.

In summary, there may be physiological differences in how children’s bodies respond to exercise as compared to adults. Therefore, the aim of this study is to investigate the difference in physiological processes between children and adults, considering submaximal and maximal parameters of CPET.

## Methods

### Subjects

For this cross-sectional study 24 children and 40 adults volunteered after written informed consent was obtained from adults and the children’s parents, respectively. Inclusion criteria for the children group were 7–11 years of age, no acute or chronic medical condition, actively involved in 2–6 h of organized sports, along with ~2–4 h of recreational physical activity. For adults, inclusion criteria were no acute or chronic medical condition and 20–30 years of age. This adult group was further divided into moderately-trained (MTA) (*N* = 20) and well-trained adults (WTA) (*N* = 20). While the MTA group consisted of recreationally active adults, the WTA group was composed of sports students. The purpose was to have one adult group that matches the cardiorespiratory fitness level of the children's group and have one group that was better trained in order to investigate the potential effects of fitness level. An additional criterion for all participants was the Physical Activity Readiness Questionnaire.^24^ If any question was answered with “Yes”, the person was excluded. The study was conducted in accordance with the principles outlined in the Declaration of Helsinki and received approval from the local ethics commission (EKNZ, Project-ID 2023-00181).

### Experimental protocol

The study consisted of one study visit. Participants were instructed to ensure adequate nutrition and hydration, with the last substantial meal not occurring within 2 h prior to the assessment. It was also emphasized that participants should refrain from engaging in exhaustive or intensive training on the day preceding the measurement. On the test day, participants underwent anthropometric measurements. In detail, body and sitting height were measured and bioelectrical impedance analysis was used to determine body composition and body mass (Inbody 720, Biospace Co., Ltd., Seoul, Korea). Furthermore, in the children group, the Mirwald method was utilized to calculate the deviation from peak height velocity as an indicator for biological maturity.^25^ Subsequently, a cardiopulmonary exercise test was performed on a cycle ergometer until exhaustion using a ramp protocol. To account for the children’s height, a smaller pediatric ergometer (Corival Pediatric, Lode B.V. Medical Technology, Groningen, Netherlands) was used instead of the adult ergometer (Excalibur Sport, Lode B.V. Medical Technology, Groningen, Netherlands). There were five different ramp protocols which started at 7, 10, 20, 50 and 50 W with a steady-state warm-up on the respective intensity. This workload was then increased until exhaustion by 7, 10, 15, 20, or 30 W/min, respectively. To reach maximal exhaustion between 8 and 10 min, the ramp protocols were chosen depending on the predicted workload according to the age and mass of the participants.^26,27^

The participants were advised to maintain a stable pedalling frequency between 60 and 80 revolutions per minute. They were verbally pushed to perform at their best to reach maximal exhaustion, and if 60 revolutions per minute could no longer be maintained despite strong encouragement, the test was terminated. Rate of perceived effort was asked every two min and after the test using the validated Pictorial Children’s Effort Rating Table for both age groups.^28^

Respiration and gas exchange were measured breath-by-breath with a gas analyser (MetaMax® 3B, Cortex Biophysik GmbH, Leipzig, Germany). Heart rate was continuously monitored using a twelve-lead electrocardiography (Custo med GmbH, Ottobrunn, Germany). The gas and volume sensors were calibrated before each measurement.

### Data processing

All respiration parameters were smoothed with a moving average of 30 s. From the original sample of 64 participants, nine were excluded from the analysis, due to measurement artifacts likely caused by the volume or gas sensor or poor data quality probably due to an unstable pedaling frequency.

VO_2_ plateaus for both, children and adults were calculated using the same approach as in Niemeyer et al.^10^, as this considers for the expected increase in VO_2_ related to the ramp protocol. A plateau was identified if the increase in VO_2_ was below <50% of the expected increase between the last and the second-to-last minute of the CPET. The expected increase in VO_2_ was calculated based on the assumption that VO_2_ increases 10 mL/min/W for each participant.

For adults, the established secondary exhaustion criterion of RER_max_ ≥ 1.13 was used.^12^ Since no validated criteria currently exist for children, we used the commonly employed threshold of HR_max_ ≥ 95% of 195 beats per min.^13^ If those criteria were met, they were considered as exhausted and included in all analysis. If not, these participants were only included in the analysis of submaximal parameters. In the present analysis, three adults and one child did not fulfil the respective criteria.

The VT1 and VT2 were set considering the V’O_2_/V’CO_2_ panel, V’E/V’O_2_ and V’E/V’CO_2_, as well as PetO_2_ and PetCO_2_ (end-tidal partial pressure). Two experienced researchers set the thresholds individually, before finding consensus. OUE in the CPET was represented by dividing VO_2_ by VE during the whole test. The OUEP was defined as the highest 90 s average of the consecutive OUE values. The overall OUES was calculated by employing a linear least squares regression of VO_2_ on the common logarithm of VE, encompassing all exercise data.^22^

### Statistics

Descriptive statistics, including the mean and standard deviation were employed to summarize the characteristics of all variables. To examine the age and sex differences, the following outcome variables were considered: VO_2_@VT1 [mL/kg/min], VO_2_@VT2 [mL/kg/min], VO_2_@VT1/VO_2peak_ [%], VO_2_@VT2/VO_2peak_ [%], OUES [mL/min], OUES_rel_ [mL/kg/min], OUEP [mL/min], OUEP/VO_2peak_ [%], OUE@VT1 [mL/min], OUE@VT2 [mL/min], O_2_/P (Efficiency), P@VT1/P_peak_ [%], P@VT2/P_peak_ [%].

Linear regression models were employed with sex and age category as covariates to examine group differences. To visualize the normal distribution, Q-Q plots were considered. Meaningful differences were interpreted considering effect sizes (Cohen’s d). Generally, effects within the range of 0.2–0.5 were categorized as small, those from 0.5 to 0.8 as medium, and values exceeding 0.8 are deemed large. Statistical significance was predetermined at an alpha level of *p* < 0.05. All statistical analyses and figures were conducted using RStudio (version 2023.12.1 + 402) with the following packages: ggplot2, lme4.^29^

## Results

**Fig. 1:**
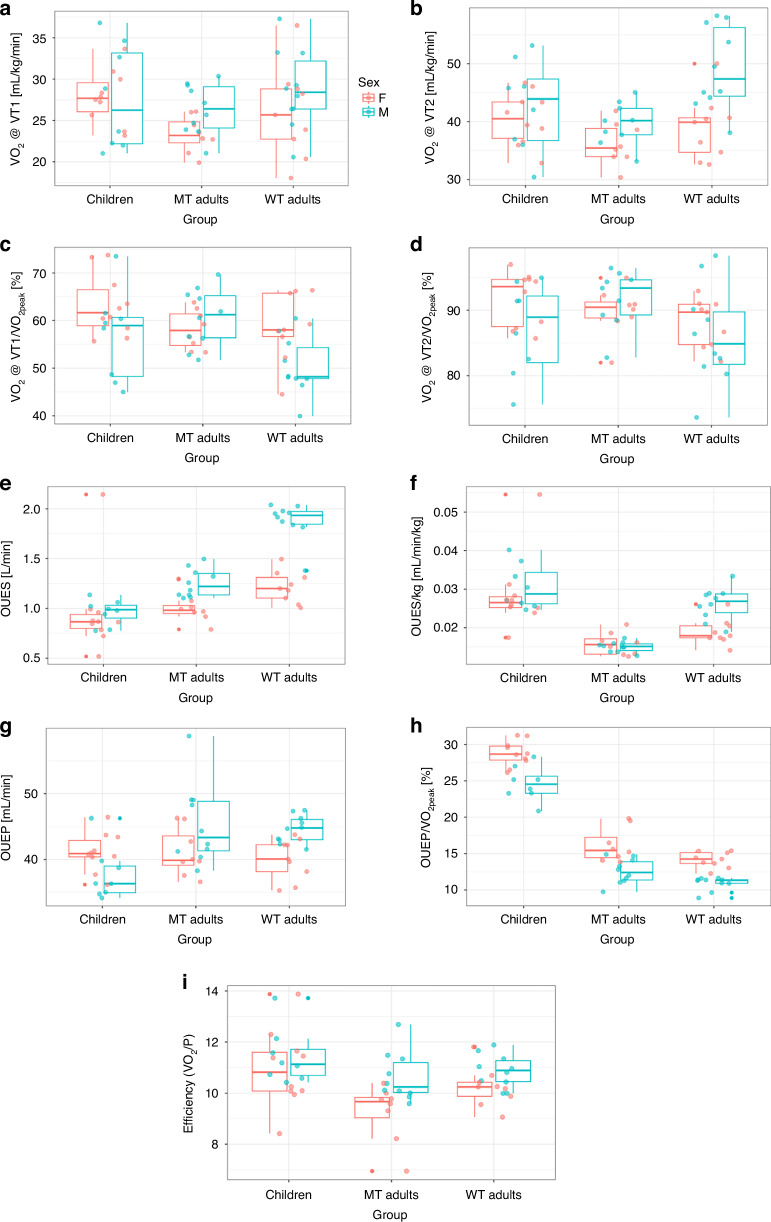
Cardiorespiratory exercise performance parameters across groups and sexes. Boxplots display key CPET-derived variables for children, moderately trained (MT) adults, and well-trained (WT) adults, with females in red and males in blue. **a** VO_2_ at ventilatory threshold 1 (VT1), (**b**) VO_2_ at ventilatory threshold 2 (VT2), (**c**) VO_2_ at VT1 relative to VO_2peak_ (VO_2_@VT1/VO_2peak_), (**d**) VO_2_ at VT2 relative to VO_2peak_ (VO_2_@VT2/VO_2peak_), (**e**) Oxygen uptake efficiency slope (OUES), (**f**) OUES relative to VO_2peak_ (OUESrel), (**g**) Oxygen uptake efficiency plateau (OUEP), (**h**) OUEP relative to VO_2peak_ (OUEP/VO_2peak_), (**i**) Exercise efficiency expressed as the ratio of VO_2_ to power output (O_2_/P).

Descriptive statistics for anthropometric characteristics, years to peak height velocity, and VO_2peak_ are presented in Table 1 for each group: children, MTA and WTA with each group further subdivided into male and female sex. Table 2 presents the descriptive CPET parameters for each group, categorized by sex. All parameters are depicted in individual boxplots, labeled accordingly (Fig. 1 and supplementary Fig. S1): VTs (A–D) and efficiency parameters (E–M). The detailed models featuring estimates, standard errors, confidence intervals, Cohen’s d values, and corresponding significance levels for the parameters are outlined in supplementary table S1. Supplementary table S2 outlines the exhaustion criteria for the CPET within the three groups and the total number of VO_2_ plateaus achieved in each group.

**Table 1:** Descriptive statistic of participants characteristics with anthropometric and CPET parameters in children, moderately-trained and well-trained adults presented in mean (SD), each separated for both sexes.

	Children (C)			Moderately-trained adults (MTA)			Well-trained adults (WTA)		
	both sexes	female	male	both sexes	female	male	both sexes	female	male
*N*	18	10	8	18	8	10	19	8	11
Age [years]	9.4 (0.9)	9.8 (0.6)	8.9 (0.9)	25.1 (2.8)	23.3 (2.0)	26.6 (2.4)	24.7 (1.6)	24.4 (1.6)	25.0 (1.7)
Height [cm]	139.4 (5.5)	142.1 (4.4)	136.1 (5.2)	173.5 (9.2)	165.9 (8.2)	179.5 (3.8)	173.8 (10.9)	165.8 (4.8)	181.0 (9.9)
Body mass [kg]	32.3 (3.8)	32.7 (3.2)	31.9 (4.7)	74.9 (12.2)	64.2 (7.3)	83.5 (7.3)	68.9 (8.2)	64.9 (6.9)	72.5 (7.9)
Fat-free mass [kg]	27.3 (3.4)	26.7 (1.3)	27.9 (4.9)	58.7 (11.6)	46.8 (2.9)	68.3 (4.2)	56.6 (10.7)	48.8 (5.9)	62.8 (9.6)
Skeletal muscle mass [kg]	14.1 (1.7)	13.8 (0.8)	14.4 (2.5)	33.1 (7.2)	25.7 (1.7)	39.1 (2.6)	32.0 (6.6)	27.3 (3.8)	35.8 (5.9)
PHV-offset	–2.66 (0.5)	–2.51 (0.4)	–2.87 (0.6)						
Relative VO_2peak_ [mL/kg/min]	45.4 (6.6)	42.6 (5.6)	48.8 (6.5)	41.9 (4.2)	39.9 (3.3)	43.5 (4.3)	50.2 (6.5)	45.9 (5.1)	54.1 (5.2)
Absolute VO_2peak_ [L/min]	1.48 (0.13)	1.43 (0.07)	1.54 (0.18)	3.16 (0.69)	2.57 (0.25)	3.64 (0.48)	3.50 (0.72)	2.83 (0.19)	4.11 (0.38)

**Table 2:** CPET parameter outcomes in children, moderately-trained and well-trained adults presented in mean (SD), each separated for both sexes

	Children (C)			Moderately-trained adults (MTA)			Well-trained adults (WTA)		
	both sexes	female	male	both sexes	female	male	both sexes	female	male
VO_2_ at VT1 [mL/kg/min]	27.8 (4.7)	27.8 (3.2)	27.7 (6.3)	25.0 (3.1)	23.3 (2.2)	26.4 (3.1)	27.4 (5.2)	26.0 (5.5)	28.8 (4.8)
VO_2_ at VT2 [mL/kg/min]	41.5 (6.1)	40.5 (4.6)	42.7 (7.8)	38.0 (4.0)	36.1 (3.7)	39.6 (3.7)	44.3 (8.2)	38.9 (5.5)	49.2 (7.2)
VO_2_ at VT1/VO_2peak_ [%]	60.3 (8.3)	63.2 (6.4)	56.7 (9.4)	59.5 (5.3)	58.1 (4.1)	60.6 (6.1)	54.2 (7.7)	58.5 (7.2)	50.3 (6.0)
VO_2_ at VT2/VO_2peak_ [%]	89.7 (5.9)	91.7 (4.2)	87.1 (7.1)	90.9 (4.1)	89.8 (3.8)	91.8 (4.4)	87.1 (6.0)	88.1 (3.7)	86.2 (7.6)
OUES [L/min]	0.96 (0.33)	0.96 (0.44)	0.96 (0.13)	1.14 (0.19)	1.00 (0.15)	1.25 (0.14)	1.56 (0.38)	1.21 (0.16)	1.88 (0.19)
OUES_rel_ [mL/min/kg]	29.5 (8.1)	28.6 (9.8)	30.6 (5.8)	15.4 (2.1)	15.8 (2.9)	15.0 (1.3)	22.8 (5.3)	18.9 (3.4)	26.2 (4.3)
OUEP [mL/min]	39.6 (3.8)	41.1 (2.9)	37.7 (4.0)	43.5 (5.5)	41.1 (3.7)	45.3 (6.1)	42.4 (3.5)	40.6 (2.9)	43.8 (3.3)
OUEP/VO_2peak_ [%]	26.9 (2.9)	28.8 (1.8)	24.6 (2.3)	14.2 (2.7)	16.2 (2.3)	12.6 (1.7)	12.5 (1.9)	14.2 (1.1)	10.9 (0.9)
O_2_/Watt [mL/min/Watt]	11.2 (1.3)	10.9 (1.5)	11.4 (1.1)	10.0 (1.2)	9.2 (1.1)	10.6 (1.0)	10.6 (0.8)	10.2 (0.8)	10.9 (0.7)
OUE at VT1 [mL/min]	38.8 (3.9)	40.2 (4.0)	37.1 (3.1)	43.5 (5.7)	41.9 (4.9)	44.8 (6.2)	42.3 (4.0)	39.7 (3.9)	44.6 (2.3)
OUE at VT2 [mL/min]	31.1 (3.1)	31.7 (2.5)	30.3 (3.7)	34.2 (3.3)	34.0 (3.0)	34.3 (3.7)	34.2 (3.7)	32.9 (3.4)	35.4 (3.8)
P at VT1/P_peak_ [%]	50.3 (10.5)	53.1 (8.1)	46.9 (12.6)	49.8 (7.7)	47.9 (7.6)	51.3 (7.9)	47.5 (8.3)	50.4 (8.4)	44.9 (7.8)
P at VT2/P_peak_ [%]	84.8 (6.7)	88.0 (3.9)	80.2 (7.4)	87.5 (5.0)	85.8 (5.8)	89.1 (3.8)	82.7 (6.8)	82.9 (5.6)	82.4 (8.0)

*VT1* Ventilatory threshold 1, *VT2* Ventilatory threshold 2, *OUE* Oxygen Uptake Efficiency, *OUES* Oxygen Uptake Efficiency Slope, *OUEP* Oxygen Uptake Efficiency Plateau, *P* Power.

Figure 1 and Supplementary Table S1 illustrate that, following CPET, children achieved moderately higher values for both VO_2_@VT1 and VO_2_@VT2 compared to MTA. In contrast, the difference between children and WTA was not as pronounced. For VO_2_@VT1, children achieved slightly higher values without significant effects, whereas WTA reached slightly higher values for VO_2_@VT2. No notable difference was observed between children and MTA when VO_2_@VT1 was considered relative to VO_2peak_. However, children showed moderately higher values compared to WTA. A difference was observed between children and MTA in VO_2_@VT2 relative to VO_2peak_, with MTA reaching moderately higher values. Meanwhile, there was a small difference between children and WTA, with children again showing higher values regarding VO_2_@VT2 relative to VO_2peak_.

Children reported a slightly lower OUES compared to MTA, and clearly a lower OUES compared to WTA. When expressed relative to body weight, the results reversed, with children having higher OUES_rel_ values compared to both adult groups. The OUEP was higher in both MTA and WTA compared to children, whereas in MTA it was highest. In contrast, when OUEP was considered relative to VO_2peak_, children exhibited substantially higher values than both WTA and MTA. Additionally, the O_2_/P ratio in children was higher compared to both adult groups.

When considering sex differences, males demonstrated higher absolute values for both VO_2_@VT1 and VO_2_@VT2 compared to females. In contrast, when VO_2_@VT1 and VO_2_@VT2 were expressed relative to VO_2peak_, females exhibited higher values than their male counterparts. Furthermore, males displayed higher values than females for OUES, OUES_rel_, OUEP, and O_2_/P.

## Discussion

The aim of this study was to investigate the differences in physiological responses during exercise between children, MTA and WTA by examining VTs and OUE during CPET. The primary findings indicate that children displayed higher absolute VT1 and VT2 values compared to MTA, while showing similar VT1 values and lower VT2 values to WTA. Additionally, adults exhibited higher OUES and OUEP values compared to children.

### Comparison between children and adults

#### Ventilatory thresholds

Our finding of children displaying higher VO_2_ at VT1 during the CPET than MTA is consistent with the results reported by previous studies.^17,18^ Furthermore, we showed that, similar to VO_2_ at VT1, children demonstrate higher VO_2_ at VT2 values compared to MTA, which is physiologically plausible, because pre-pubertal children rely more on oxidative metabolism for energy production in engaged muscles compared to adolescents and adults.^30^ A higher proportion of oxidative metabolism would consequently result in a lower proportion of anaerobic metabolism. Although this study did not measure this directly, previous studies have demonstrated lower anaerobic capacity in children, which increases as they mature.^31^

To explain physiological differences between children and adults, Dotan et al.^32^ proposed that children’s elevated oxidative and reduced anaerobic capacity result from a lower recruitment of large motor units innervating type II muscle fibers.^32^ While skeletal muscle biopsy studies investigating fiber type distribution remain sparse in children, this hypothesis serves as an explanation for the higher VO_2_ at VT1 and VO_2_ at VT2 observed in children. In line with this fiber recruitment, the activity level of oxidative enzymes have been shown to be higher in children highlighting the greater oxidative potential at younger age.^30,33,34^ Therefore, children’s greater oxidative yet diminished anaerobic capacity in muscle fibers^4^ may account for the higher VO_2_ at VT1 observed in children compared to MTA, likely resulting from a reduced accumulation of metabolic by-products (such as H+ ions, lactate, and inorganic phosphate). These by-products stem from anaerobic processes in exercising muscles, are linked to the pH, and may therefore also account for the higher VO_2_ at VT2.^35^ Additionally, a previous study has shown that children exhibit a higher density of mitochondria. This higher density enables more efficient oxygen utilization, resulting in greater aerobic capacity.^36^ However, as mitochondrial density declines with age,^30^ the efficiency of oxygen utilization decreases, leading to a reduction in aerobic capacity and a shift in metabolism towards anaerobic processes, which could also explain the lower VO_2_ at VT1 and VO_2_ at VT2 values in MTA compared to children.

On the other hand, the higher VO_2_ at VT2 could also reflect a better fitness level, as WTA displayed higher values than children and MTA. Indeed, previous research demonstrated that the higher VTs might be attributed to the higher engagement in endurance-based training, which could counteract the decline in aerobic capacity with age^37^ and therefore could result in higher VO_2_ at VT1 and VO_2_ at VT2 in WTA compared to MTA.

It has been demonstrated that individuals with higher anaerobic capacity can maintain increased workloads beyond VO_2max_, which may lead to the occurrence of a VO_2_ plateau.^10^ This is reflected in our study, where a higher frequency of VO_2_ plateaus was observed in MTA compared to children (see Supplementary Table S2). Finally, due to higher mitochondrial density and oxidative enzyme activity, children demonstrate a greater phosphocreatine recovery rate (PCr) than adults.^30^ This faster PCr recovery indicates again a more oxidative, less glycolytic metabolic profile.^32^

When analyzing the VTs relative to VO_2peak_, VT1 as a percentage of VO_2peak_ does not differ between children and MTA, while WTA demonstrate lower values compared to both groups (see Fig. 1c). For VT2/VO_2peak_ (see Fig. 1d), MTA exhibit higher values than children, while WTA show the lowest values. This can be partly explained by the VO_2peak_ differences, with WTA having the highest VO_2peak_, followed by children, while MTA display the lowest VO_2peak_ values (Table 2), which affects the ratio.

#### Oxygen uptake efficiency

Our results indicate that adults exhibit higher absolute OUES values compared to children. This finding aligns with previous studies, as both Marinov et al.^38^ [CIT] and Bongers et al.^22^ indicate that absolute OUES values increase with age. However, the latter study only compared children with adolescents and did not include adults. Similar results were also shown by Rogowski et al.^23^, who found that absolute OUES values were lower in less mature groups compared to more mature ones. The increased ventilation needed by younger individuals to expel CO_2_ and maintain lower PaCO_2_ levels^39^ may explain the lower absolute OUES values seen in younger populations.^23^ As expected, WTA displayed higher OUES values compared to MTA, which is in line with previous research showing that OUES tends to increase following physical training.^40^

To account for anthropometric differences among participants, OUES can be standardized relative to body mass, body surface area, or fat-free mass.^1^ Rogowski et al.^23^ demonstrated that when adjusted for body mass, OUES_rel_ values were higher in less mature individuals compared to their more mature counterparts. Consistent with this, our findings revealed that OUES_rel_ was higher in children compared to adults. This indicates that OUES_rel_ differences are likely more influenced by maturation than by body mass and are therefore possibly associated with metabolic and physiological changes. Factors such as metabolic acidosis, arterial CO_2_ set point and CO_2_ production^21^ are affected by the balance between glycolytic and oxidative metabolism during exercise. Since this metabolic balance shifts with maturation, the variations in OUES between children and adults are probably due to these underlying physiological and metabolic differences rather than body size alone.^23^ Indeed, studies have shown that established differences in glycolytic and oxidative metabolism between children and adults likely impact the OUES mediators and variables (VO_2_ and VE).^41^

Additionally, our results demonstrate that adults exhibit higher OUEP values compared to children, which is consistent with the findings of Bongers et al.^22^. OUEP values were also slightly higher in WTA compared to MTA. This is consistent with the findings of Sun et al.^42^, who demonstrated that OUEP is minimally influenced by fitness level. The OUEP represents the highest efficiency of an individual’s oxygen uptake relative to their ventilation.^42^ When comparing different age groups, it is observed that children have lower OUEP values compared to adults, indicating that children are less efficient and require more VE to achieve the same level of VO_2_.^22^ This inefficiency in younger children can be attributed, as with OUES, to their higher ventilatory requirements to effectively eliminate CO_2_ and maintain appropriate arterial CO_2_ levels.^21^ As a result, the ratio of oxygen uptake to ventilation is less efficient in children compared to older individuals. In contrast, adults generally have higher OUEP values due to their more mature and efficient systems for oxygen transport and utilization during exercise.^43^

However, upon examining the OUEP in relation to VO_2peak_, we find that children demonstrate a higher relative performance compared to adults. This elevated ratio might indicate that, although children have a lower absolute efficiency in oxygen uptake (reflected in their lower OUEP values) they may utilize oxygen more effectively relative to their maximum aerobic capacity. Consequently, this suggests that children might achieve a higher level of individual performance, potentially highlighting developmental differences in cardiorespiratory function.

### Comparison between male and female

#### Ventilatory thresholds

Reybrouck et al.^18^ reported that girls reached VT1 at a significantly lower VO_2_ than boys of the same age, which contrasts with our findings, where boys exhibited almost equal VO_2_ at VT1 values as girls. However, in MTA and WTA, males displayed higher VO_2_ at VT1 values than females, consistent with the sex-specific trend reported by Reybrouck et al.^18^. Additionally, our results show that males across all groups had higher VO_2_ at VT2 values compared to females, aligning with previous studies indicating that anaerobic metabolism begins at lower exercise intensities in female than in male children.^18^ Cooper et al.^17^ further highlighted sex differences, showing that girls tend to have lower VO_2peak_ and VT1 values compared to boys, potentially due to a higher percentage of body fat. However, in our study, the body fat percentage between boys and girls was quite similar, suggesting that other factors, such as differing behaviors in physical activity, movement experience^44^ and variations in biological maturation between boys and girls, may contribute to the observed differences, and further investigation is needed to draw definitive conclusions.

Our results also indicate that females demonstrated higher VT1/VO_2peak_ and VT2/VO_2peak_ ratios in both children and WTA compared to males, likely due to the higher VO_2peak_ values observed in male children and WTA. In contrast, in the MTA group, male participants compared to female participants showed higher VT1/VO_2peak_ and VT2/VO_2peak_ ratios, which can be attributed to their comparatively lower VO_2peak_ values. Interestingly, the difference in VT1/VO_2peak_ between moderately-trained and well-trained male adults is substantial, while this difference is minimal between moderately-trained and well-trained female adults. This may suggest sex-specific adaptations to endurance training, possibly due to differences in muscle fiber distribution, with men having a higher proportion of type II fibers,^45^ which are associated with greater anaerobic capacity. As a result, training may have a more pronounced effect in men, potentially explaining the observed differences. This hypothesis warrants further investigation.

#### Oxygen uptake efficiency

Bongers et al.^22^ demonstrated that OUES values increase with age in both boys and girls, with boys exhibiting higher values. This sex-specific trend aligns with our findings, which show that OUES values are higher in adults compared to children, with males achieving greater values.

The OUEP shows a significant increase from boys to men, whereas it remains relatively constant in females across age groups. This finding contrasts with the results of Bongers et al.^22^, who reported no sex differences and suggested that OUEP increases similarly in both sexes. However, Bongers et al.^22^ did not take other relevant factors, such as maturation and anthropometrics, into account, which could explain the different results. In our study, the observed sex differences in children could potentially be attributed to the fact that the girls in our study were slightly older or more mature at the time of testing, which may have influenced their OUEP values.

## Limitations

The primary limitation of this study lies in the cross-sectional design, which precludes the establishment of causality between growth, development, and the outcomes. Longitudinal studies would provide a more comprehensive understanding of the effects of growth and development as well as the influence of training. Due to the elevated level of physical activity among our pediatric population, as evidenced by their relatively high VO_2peak_, these subjects may be highly trained and therefore might not be representative of a typical pediatric population. Additionally, it is important to highlight that due to the lack of robust secondary criteria, it is possible that not all included children reached maximal exertion. As we did not perform a familiarization session, the novel exercise for the children, which required maximal effort, could have diminished data quality.

## Conclusion

The mechanisms underlying the physiological and metabolic differences between children and adults remain only partially understood. It appears that children demonstrate a greater reliance on oxidative metabolism compared to adults, which may be reflected in higher absolute VO_2_ at VT1 and VT2 values. This decline in VTs from childhood to adulthood can potentially be mitigated through endurance training. Further, OUES is affected by age, as children show lower absolute OUES values as compared to adults. Interestingly, when adjusted for body mass, the opposite was true, with children showing higher OUES_rel_ values, indicating that maturation influences efficiency more than body size, reflecting underlying metabolic and physiological differences. In summary, OUEP remains a relatively understudied marker and longitudinal data are urgently needed to understand the causality and mechanisms of maturation on physiological and metabolic changes in response to exercise.

## Supplementary information

Supplementary Material_Table1

Supplementary Material_Table2_figure1

## Data Availability

The data that support the findings of this study are available on request from the corresponding author. The data are not publicly available due to ethical restrictions.
