# Supplementary material for: Physiological differences in cardiopulmonary exercise testing between children and adults
Source: Pediatr Res. 2025 Jun 19;99(2):783–90. doi: 10.1038/s41390-025-04212-9 (PMC12956600; doi:10.1038/s41390-025-04212-9)
Supplement: Supplementary file 2 — Supplementary Material_Table2_figure1 [file 41390_2025_4212_MOESM2_ESM.pdf]

Supplemental table S2: exhaustion criteria in children, moderately-trained and well-trained adults presented in mean (SD), each separated for both sexes. RER: Respiratory Exchange Ratio. HF: Heart Frequency.

	**Children (C)**			**moderately-trained adults (MTA)**			**well-trained adults (WTA)**		
Number of VO2 Plateaus	2	2	0	9	2	7	5	1	4
RER_{max}	1.12 (0.05)	1.10 (0.04)	1.14 (0.06)	1.17 (0.04)	1.16 (0.05)	1.17 (0.04)	1.20 (0.04)	1.18 (0.03)	1.23 (0.04)
HF_{max}	190.9 (8.0)	189.6 (7.4)	192.6 (8.9)	185.7 (7.5)	183.5 (5.9)	187.4 (8.4)	188.2 (5.8)	186.3 (4.6)	189.9 (6.3)

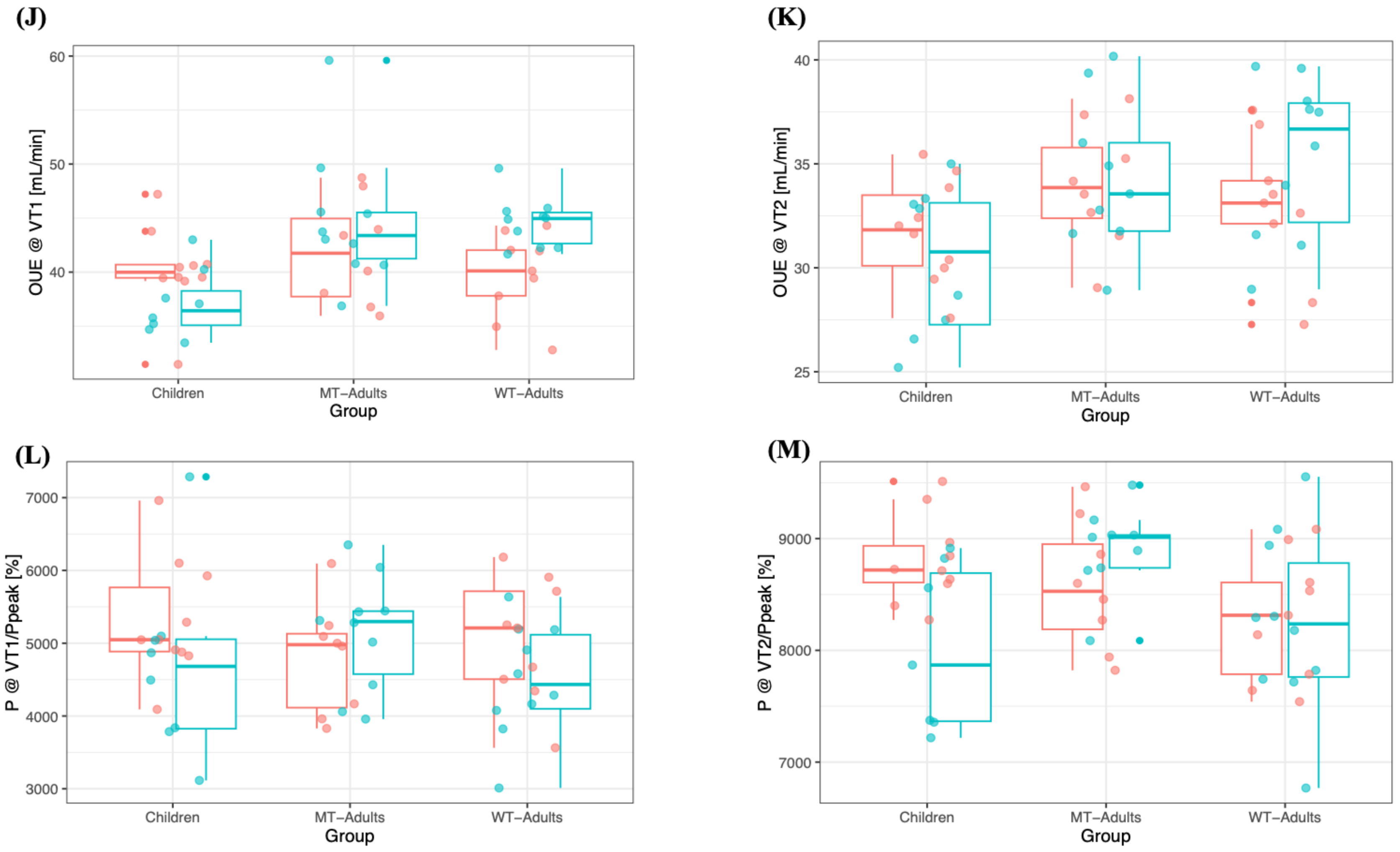

Supplemental figure 1: The supplemental CPET parameters OUE@VT1 (J), OUE@VT2 (K), P@VT1/Ppeak (L) and P@VT2/Ppeak (M) are shown in the corresponding graphs as boxplots across the three groups: children, moderately-trained, and well-trained adults. Female subjects are represented in red, while male subjects are presented in blue.
